# Genetic Depletion of BDNF Impairs Extinction Learning of a Spatial Appetitive Task in the Presence or Absence of the Acquisition Context

**DOI:** 10.3389/fnbeh.2021.658686

**Published:** 2021-04-30

**Authors:** Marta Méndez-Couz, Beate Krenzek, Denise Manahan-Vaughan

**Affiliations:** Department of Neurophysiology, Medical Faculty, Ruhr University Bochum, Bochum, Germany

**Keywords:** context-dependent, BDNF, extinction learning, rodent, appetitive, spatial learning, AAA paradigm, ABA paradigm

## Abstract

Brain derived neurotropic factor (BDNF) supports neuronal survival, growth, and differentiation and is involved in forms of hippocampus-dependent and independent learning, as well as hippocampus-dependent learning. Extinction learning comprises active inhibition of no-longer relevant learned information, in conjunction with a decreased response of a previously learned behavior. It is highly dependent on context, and evidence exists that it requires hippocampal activation. The participation of BDNF in memory processing is experience-dependent. For example, BDNF has been associated with synaptic plasticity needed for spatial learning, and it is involved in acquisition and extinction learning of fear conditioning. However, little is known about its role in spatial appetitive extinction learning. In this study, we evaluated to what extent BDNF contributes to spatial appetitive extinction learning in the presence (ABA) or absence (AAA) of exposure to the acquisition context. Daily training, of BDNF^+/−^-mice or their wildtype (WT) littermates, to reach acquisition criterion in a T-maze, resulted in a similar performance outcome. However, extinction learning was delayed in the AAA, and impaired in the ABA-paradigm compared to performance in WT littermates. Trial-by-trial learning analysis indicated differences in the integration of the context into extinction learning by BDNF^+/−^-mice compared to WT littermates. Taken together, these results support an important role for BDNF in processes that relate to information updating and retrieval that in turn are crucial for effective extinction learning.

## Introduction

Operant behaviors are voluntary actions controlled by their consequences. Animals readily acquire behaviors (e.g., lever pressing) to obtain a desirable outcome (e.g., food pellet or drug delivery) and equally learn to suppress or diminish this behavior when the reinforcer is withdrawn, in a process known as extinction learning (Eddy et al., 2016). Extinction learning of instrumental responding is a central point of behavioral change (Todd et al., 2014; Bouton, 2019). In opposition, extinguished behavior, the associated responses for which have been abolished, can re-emerge through several mechanisms. One of these processes is referred to as renewal, which occurs in the form of a return of the associated response, when an animal is tested in a context different from the one in which extinction learning took place, or when substantial time has elapsed since extinction learning occured (Bouton and Bolles, 1979). extinction learning is accelerated when animals are exposed to a context that differs from the original acquisition context, referred to here as an ABA paradigm, compared to extinction learning in the original acquisition context (AAA paradigm) (Bouton and Bolles, 1979; Bouton, 2004, 2019; Bouton et al., 2006). This reappearance of responding demonstrates that extinction learning does not comprise erasure of the original learning. Evidence exists that extinction learning may be considered to be a new form of learning, involving memory formation whilst preserving the original memory trace (Mendez-Couz et al., 2019).

Although in mechanistic studies of extinction learning the primary focus has been placed on aversive forms, studies of appetitive forms of extinction learning in rodents have offered novel insights as to the cognitive challenges, brain structures, and molecular systems involved in this process. Where extinction learning of a spatial appetitive task was studied, it was found that extinction learning in the absence of a context change requires many more task exposures and increased catecholaminergic contribution compared to extinction learning conducted in a different context (André et al., 2015a). Extinction learning in the absence of a context change also depends on activation of the metabotropic glutamate receptor, mGlu5, whereas context-dependent extinction learning does not (André et al., 2015a). Context-dependent extinction learning recruits information processing in the hippocampus that involves gene encoding (Mendez-Couz et al., 2019), a process that has been linked to hippocampal synaptic plasticity (Kemp et al., 2013) and context-dependent spatial learning (Hoang et al., 2018).

One very important mediator of signaling pathways related to gene encoding and synaptic plasticity in the brain is brain-derived neurotrophic factor (BDNF). It supports neuronal survival as well as functional and structural synaptic plasticity (Zagrebelsky and Korte, 2014). It has been proposed that activity-dependent secretion of BDNF may support synapse-specific synthesis of proteins that are required for the stability of long-term forms of synaptic plasticity (Lucidi-Phillipi et al., 1995; Poo, 2001; Novkovic et al., 2015). Moreover, secreted BDNF is capable of mediating many activity-dependent processes in the mammalian brain, including neuronal differentiation and growth, synapse formation and plasticity, and higher cognitive functions (Park and Poo, 2013), including spatial learning (Aarse et al., 2016). This is especially notable, given that synaptic plasticity in the hippocampus, in the forms of long-term potentiation and long-term depression, comprises the primary cellular mechanism underlying long-term spatial memory (Kemp and Manahan-Vaughan, 2007).

Brain-derived neurotrophic factor has been strongly implicated in clinical depression and cognitive impairments observed in depressed patients (Brunoni et al., 2008). BDNF levels can be significantly influenced by exposure to stress (Bath et al., 2013), a process that may take place during fear conditioning learning and aversive forms of extinction learning. In line with this, BDNF contributes to extinction learning of fear conditioned memory (Peters et al., 2010; Rosas-Vidal et al., 2018; Notaras and van den Buuse, 2020). Specifically, the role of BDNF has been scrutinized in studies of malfunctioning contextual fear conditioning and disrupted extinction learning, where BDNF was infused into the hippocampus (Kirtley and Thomas, 2010) or the infralimbic cortex (Peters et al., 2010). Antagonism of BDNF in the prefrontal cortex in an animal model of aversive extinction learning results in impairment of extinction learning recall and changes in ventral levels of BDNF (Rosas-Vidal et al., 2018). In humans, the genetic BDNF^*Val66Met*^ variant is associated with impaired extinction learning (Notaras and van den Buuse, 2020) as well as with a decreased response for fear-extinction therapies (Soliman et al., 2010).

Although the role of BDNF in extinction learning of conditioned aversive learning has been well-described (Karpova et al., 2011; Psotta et al., 2013), little is known about the role of BDNF in *appetitive* extinction learning of context-related experience. Here, we studied extinction learning of a spatial appetitive task in BDNF^+/−^ mice in the presence or absence of a context-change. We report no ostensible deficiencies in task learning in the acquisition context (AAA paradigm), but extinction learning was delayed in this paradigm in BDNF^+/−^ mice compared to their WT littermates. Furthermore, extinction learning occurring in a context that was different to the acquisition context was significantly impaired. These findings support a role for BDNF in information updating related to extinction learning under spatial appetitive circumstances.

## Materials and Methods

This study was carried out in accordance with the European Communities Council Directive of September 22, 2010 (2010/63/EU) for the care of laboratory animals. All experiments were performed according to the guidelines of the German Animal Protection Law and were approved by the North Rhine-Westphalia State Authority (Landesamt für Arbeitschutz, Naturschutz, Umweltschutz und Verbraucherschutz, LANUV, Bezirksamt, Arnsberg). Animal numbers were kept to the minimum required for biometric planning.

Male BDNF^+/−^ mice and their male wildtype (WT) littermates were used (Animal breeding facility of the Medical Faculty, Ruhr University Bochum). The strain was originally established by Korte et al. (1995), whereby one allele of the BDNF protein-coding exon was replaced by a neomycin-resistant gene surrounded by a glycerate kinase gene promotor and a polyadenylation signal. Heterozygous BDNF^+/−^ mice, and their WT littermates, were produced by crossing BDNF^+/−^ male mice with C57BL/6 female mice. Homozygote BDNF^–/–^ mice were not used as they express abnormalities including neuronal loss and retarded development (Korte et al., 1995; Zagrebelsky and Korte, 2014).

### Genotyping of the Animals by PCR

**FIGURE 1:**
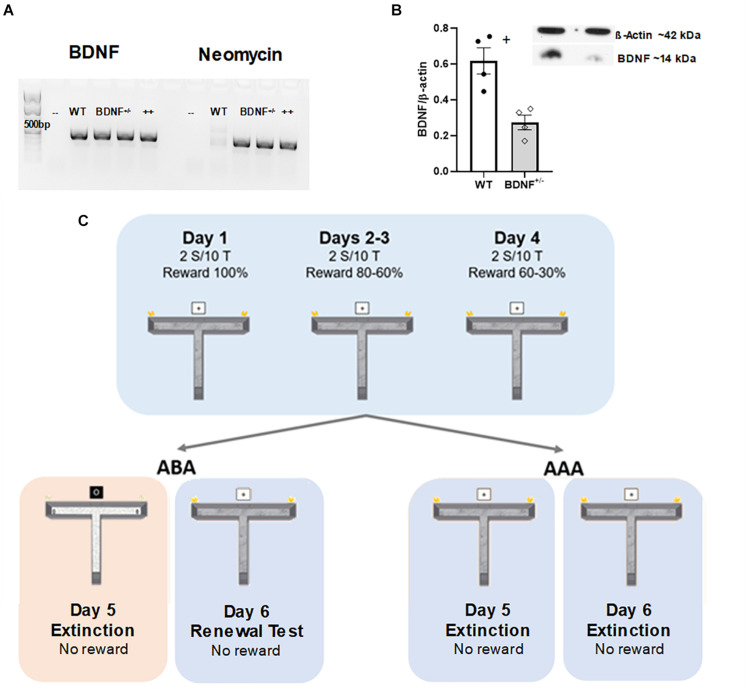
Genotyping, Western blots and schema of the behavioral protocol. **(A)** Genotyping of BDNF mice. In BDNF^+/−^ animals, a neomycin-resistant gene, surrounded by a glycerate kinase gene promotor and a polyadenylation signal, replaces one allele of the BDNF protein-coding exon. Most of the mature BDNF is therefore depleted, and the introduced gene serves as a biomarker. BDNF^+/−^ animals (bands 3 and 4, from the left in each gel) expressed both the neomycin-resistant gene (500 bp) as well as BDNF (500 bp), whereas in the wild type (WT, second band from the left) animals only the BDNF bands are observable. – and ++ bands correspond to negative and positive controls, respectively, for both genes. **(B)** Hippocampal BDNF expression is depleted in the BDNF^+/−^ animals. The expression of BDNF (14 kDa) and β-actin (42 kDa) protein in the hippocampus of both BDNF^+/−^ (*n* = 4) and wild type littermates (WT) animals (*n* = 4) was evaluated by Western blot. β-Actin was used as a protein loading control, and each individual value was normalized by the β-Actin expression level. Expression of mature BDNF was significantly lower in the transgenic animals as compared to their WT littermates. Group averages (±S.E.M) are indicated by vertical bars, points represent individual measurements (*p* = 0.006). **(C)** Schema of the behavioral protocol. Top: On Day 1, animals participate in two sessions each comprising ten consecutive trials each (separated by 10-min intervals) that include a reward probability of 100%. On Day 2 two ten trial sessions at 80% of reward probability occur. On Day 3, the reward probability of the first session is 80%, and of the second session is 60%. On Day 4, the reward probability declines from 60% in the first session, to 30% in the second session. Bottom right: On Days 5 and 6, animals participate in the extinction learning protocol in the same context (AAA) in the absence of a reward. Bottom left: In the ABA paradigm animals engage in extinction learning in the presence of a context change on Day 5. During extinction learning, the context (floor pattern, distal cues, odor cues) are changed. On Day 6, renewal is tested by re-exposure to context A. No reward is present in the maze during the extinction or renewal test days.

In this BDNF transgenic mouse strain, the inserted neomycin resistant gene serves as a biomarker for the genetic modification (Novkovic et al., 2015). To genotype the mice, a polymerase chain reaction (PCR) was performed on biopsied ear tissue (of >3 week old mice). A PCR primer (BDNF_1S 5′ -ACC ATA AGG ACG CGG ACT TGT AC-3′) was used to recognize BDNF and another primer (Neo_1S 5′ GAT TCG CAG CGC ATC GCC TT-3′) was used to detect neomycin. A reverse primer (BDNF_1AS 5′-GAA GTG TCT ATC CTT ATG AAT CGC-3′) was also used. Bands were revealed in 1.5% Agarose Gel (100 ml + 5 μl Ethidiumbromide) (Figure 1A).

### Assessment of Hippocampal BDNF by Western Blot

To analyze the amount of BDNF protein, Western blots were conducted. Brains were removed at 6–7 weeks of age. Hippocampi were manually separated and homogenized at 4°C in Tris–HCl buffer (20 mM, pH 7.4) containing 10% sucrose. Homogenates were centrifuged at 4°C for 30 min with 14,000 *g*. Pellets were resuspended in ice-cold Tris-HCl buffer, pH 7.4, containing protease inhibitors (Roche Diagnostics). The total protein concentration of each sample was determined by Bradford protein assay (Ultrospec 3000, Pharmacia Biotech) to ensure that the same total amount of protein (10 μg) was applied to the gel. The gel electrophoresis of proteins was carried out using 15% sodium dodecyl sulfate (SDS) polyacrylamide gels run on a mini-gel apparatus (Pequlab) for 1.5 h at room temperature. The Western blot transfer was conducted in a cold buffer (wet conditions) in a Western blot transfer system (Consort) at 400 V, 300 mA for 1.5 h. Membranes were incubated for 1 h in Tris-buffered-saline (100 mM Tris-HCl; 0.9% NaCl, 1% Tween 20, pH 7.4) containing 5% non-fat milk to block non-specific binding sites. Blots were then incubated overnight at 4°C with a primary monoclonal anti-rabbit antibody (1:1,000 dilution, Epitomics, UK cat. no. 3160-1) that labels mature BDNF (14 kDa). Protein β-actin was used as a loading control, labeled with a monoclonal antibody (1:20,000 Sigma, St. Louis, MO Cat no. A5316, RRID:AB_476743). The immunoreactivity was visualized using horseradish peroxidase (HRP)-coupled goat anti-rabbit secondary antibodies (1:20,000 dilution, GE Healthcare) and an enhanced chemiluminescence (ECL) detection system (GE Healthcare). The immunoblots were subsequently analyzed with ImageJ Software (NIH, Bethesda, MD, United States) to measure the optical density of the bands. BDNF expression was measured in proportion to β-actin in the hippocampus (Figure 1B).

### Behavioral Paradigm

The experimental room was faintly illuminated during experiments and animal behavior was recorded by means of a monitoring system (Videomot; TSE Systems, Bad Homburg, Germany) that enabled subsequent offline analysis. Extinction learning experiments were conducted in a T-maze that was composed of a starting box (20 cm × 20 cm) that was separated from the main corridor (100 cm × 20 cm) by a sliding door and two side corridors (40 cm × 10 cm) positioned perpendicularly to the other end of the main corridor (Figure 1). The walls were 40 cm high. In each side corridor, a small round cup was placed 1 cm in front of the end wall and in the middle of the floor, where the reward was hidden from distant view. The reward consisted of 100 μl of 25% sucrose solution. This form of reward was chosen because according to Adachi et al. (2008), the deletion of BDNF in the hippocampal CA1, or in the DG region does not alter preference for a natural reward in the sucrose preference test in comparison to controls. The maze design and the protocol followed were as described previously (Wiescholleck et al., 2014; André and Manahan-Vaughan, 2015; André et al., 2015a,b; Figure 1C).

The context of the maze was changeable by three aspects, floor pattern, external visual cues, and internal odor cues. The maze floors had distinct visual patterns such as wood-like, totally white, or granite-like patterns printed on washable PVC. During each phase of training or testing, 1 μl of a particular odor (e.g., almond or vanilla food aroma, Dr. Oetker, Bielefeld, Germany) was placed at the end of the two arms. For visual distal cues, Din A4 size cards of white paper printed with a thick black cross, or a black-filled circle, were used. These visual cues were positioned 40 cm above the end of the main corridor (Figure 1C). The visual cues remained constant in all trial conditions with the exception of extinction learning in the ABA paradigm. The same odor and visual was used throughout acquisition trials, and during all sessions of the AAA paradigm, whereby the AAA paradigm tested acquisition and extinction learning in the same context (Figure 1C). In this case, extinction learning was monitored on two consecutive days.

To test extinction learning in a different context as the one used for acquisition, an ABA paradigm was used. Here, during extinction learning the odors, floor pattern, and distal visual cues were changed for novel cues. The renewal trials were conducted under the same conditions as those used for acquisition trials (Figure 1C).

A week before the beginning of the behavioral training, the mice were weighed and food availability was reduced to achieve a minimum of 85% of the previously determined body weight. This weight was sustained until the end of the experiment.

Every day, each mouse participated in a series of trials, consisting of two sessions of 10 consecutive trials with an intertrial time of 15 s and 30 min pause between sessions. Each trial began when the door to the starting box was opened and the animal could enter the maze. It ended when the animal entered an arm of the T-maze, or when 30 s had elapsed without leaving the starting box. In each trial, the animals were expected to search for a reward that was placed in the indentation located in the floor at the end of a predetermined arm. From Days 1 through 4, the reward probability was decreased stepwise from 100 to 30% to increase extinction learning resistance. Specifically, on the first day of acquisition, all trials were rewarded (100%), on the second day, 80% of the trials were rewarded, the third day 80% of the trials were rewarded in the first session and 60% on the second session, and in the last day, 60 and 30% of the trials were rewarded in the first and last session respectively. Without this form of training, testing contextual changes during repeated extinction learning trials in the absence of a food reward would not have been possible (André et al., 2015b). Learning criterion was reached when the animal successfully entered the correct arm on eighty-five per cent of the trials in the last session. Animals that did not reach a minimum of 85% correct responses by the final trial of Day 4, were excluded from the study. In between trials, the maze was wiped with a humid cloth to mix the odor trail that the animal could have left behind. Before the next animal was exposed to the T-Maze it was thoroughly cleaned and dried.

In the case of the ABA paradigm, extinction learning was evaluated on Day 5. For this purpose, mice were introduced into the T-maze in absence of food reward for two sessions of 10 trials during which the context (floor pattern, odor, and cue card) was changed. On Day 6, renewal (RN) was assessed. Here, the animal was reintroduced to the original T-maze context (A) for another two sessions of ten trials each, but a food reward was also absent in this case.

In case of the AAA paradigm, mice underwent the same procedure in the original acquisition context (A), but during the 5th and 6th days, when extinction learning was examined, the food reward was perpetually absent.

### Statistical Analysis

For Western blot analysis, an unpaired Student’s *t*-test was used to evaluate BDNF expression in the hippocampus of WT and transgenic mice.

For the behavioral analysis, video recordings of each experimental phase were analyzed for the correct or incorrect response of the animals in each trial. To prevent bias, animals were coded so that the experimenter was unaware of the behavioral condition for each video analyzed. The percentage of correct responses per session was calculated (excluding trials in which animals did not perform) and a two-way repeated-measures (rm) analysis of variance (ANOVA) was used to determine the difference in between groups for the acquisition and the extinction-renewal phases, with “group” and “session” as factors. *Post hoc* analysis was conducted using a Holm–Sidak test, to determine the differences between individual trials. A one-way rm ANOVA were applied within-groups to test the effectiveness of extinction and renewal, with “session” as factor. Here, the Student–Newman–Keuls test was applied as a *post hoc* test. For the trial by trial analysis only correct responses were taken, excluding any alternative answer, as not leaving the starting box, going back before entering an arm or entering the incorrect arm. Sigmastat 11 (Systat) and Prisma 8 (GraphPad) software were used for the analysis and graphs.

## Results

### Genotyping and Verification of BDNF Depletion

Polymerase chain reaction was used to differentiate between BDNF transgenic and WT littermates. Here, for each animal used in the study we confirmed that BDNF^+/−^ animals expressed both the inserted neomycin-resistant gene (500 bp), as well as BDNF (500 bp), whereas in the WT animals only BDNF was detected (Figure 1A). Western blot confirmed that BDNF expression levels were significantly higher in the WT animals (*n* = 4) compared to BDNF^+/−^ mice (*n* = 4) [*t*_(6)_ = 4.081; *p* = 0.006], consistent with BDNF depletion having occured in the transgenic mice (Figure 1B).

### Depletion of BDNF Does Not Impair Context-Related Appetitive Learning

Animals learned to associate a specific T-maze arm with a low probability reward over a period of 40 acquisition trials conducted on four consecutive days (Figure 1C). The learning criteria of 85% correct arm choices was reached by Day 4 in both BDNF^+/−^ mice (*n* = 16) and their WT littermates (*n* = 16) (Figure 1C).

**FIGURE 2:**
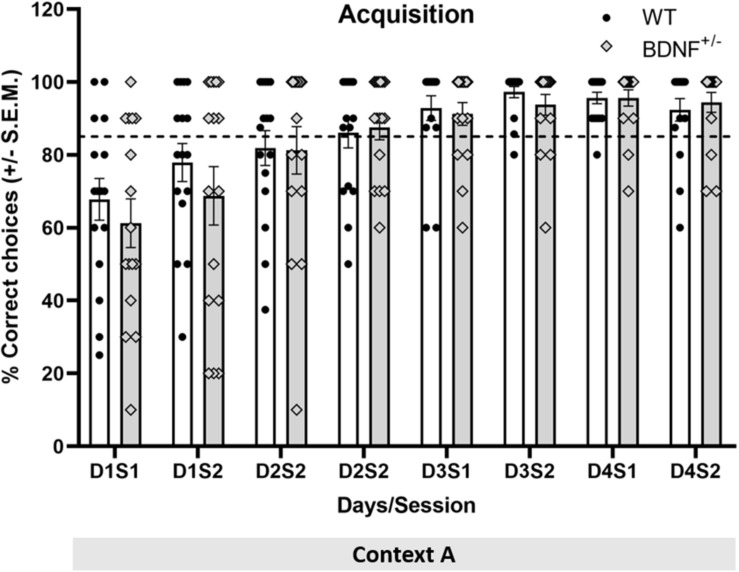
BDNF^+/−^ mice do not exhibit impairments in task acquisition. The bar charts show the percentage of correct choices made by the animals during task acquisition learning over 4 days in context A. Each bar pair shows the animals’ correct choice performance in a given session (S) on a specific day (D) of acquisition. White bars show the response of wild type (WT) mice and gray bars show the response of transgenic BDNF^+/−^ mice. The dots (black: WT, gray outlined: BDNF^+/−^) show the distribution of choice performance in a specific session. Both WT (*n* = 16) and BDNF^+/−^ animals (*n* = 16) acquired the task over the 4 days, as signified by the increasing percentage of correct responses in both groups. Despite the gradual reduction in the reward probability, the two groups reached the 85% criterion of correct responses (dashed line) by day 4.

No acquisition learning differences were found during days 1–4 between BDNF^+/−^ and WT mice [*F*_(1,210)_ = 0.25, *p* = 0.62]. Animals exhibited equivalent improvements in choice behavior between acquisition sessions [*F*_(7,210)_ = 22.84, *p* < 0.01] (Figure 2).

To further clarify if the transgenic and WT animals exhibited differences in acquisition in the AAA or ABA paradigms, a two-way repeated-measures ANOVA was carried out for the percentage of correct decisions taken, with session and group used as factors. No statistically significant interaction between the factors session and group was evident i.e., the effect of the performance in the different test groups did not differ depending on the session tested [*F*_(21,196)_ = 0.61, *p* = 0.91]. Similarly, no statistically significant difference between groups was found in terms of the percentage of correct trials along acquisition sessions [*F*_(3,196)_ = 1.05, *p* = 0.38]. Taken together, this indicates that both transgenics and WT animals acquired the task equally effectively and at the same learning pace.

### Extinction Learning Is Delayed in BDNF^+/−^ Mice in the AAA Paradigm

**FIGURE 3:**
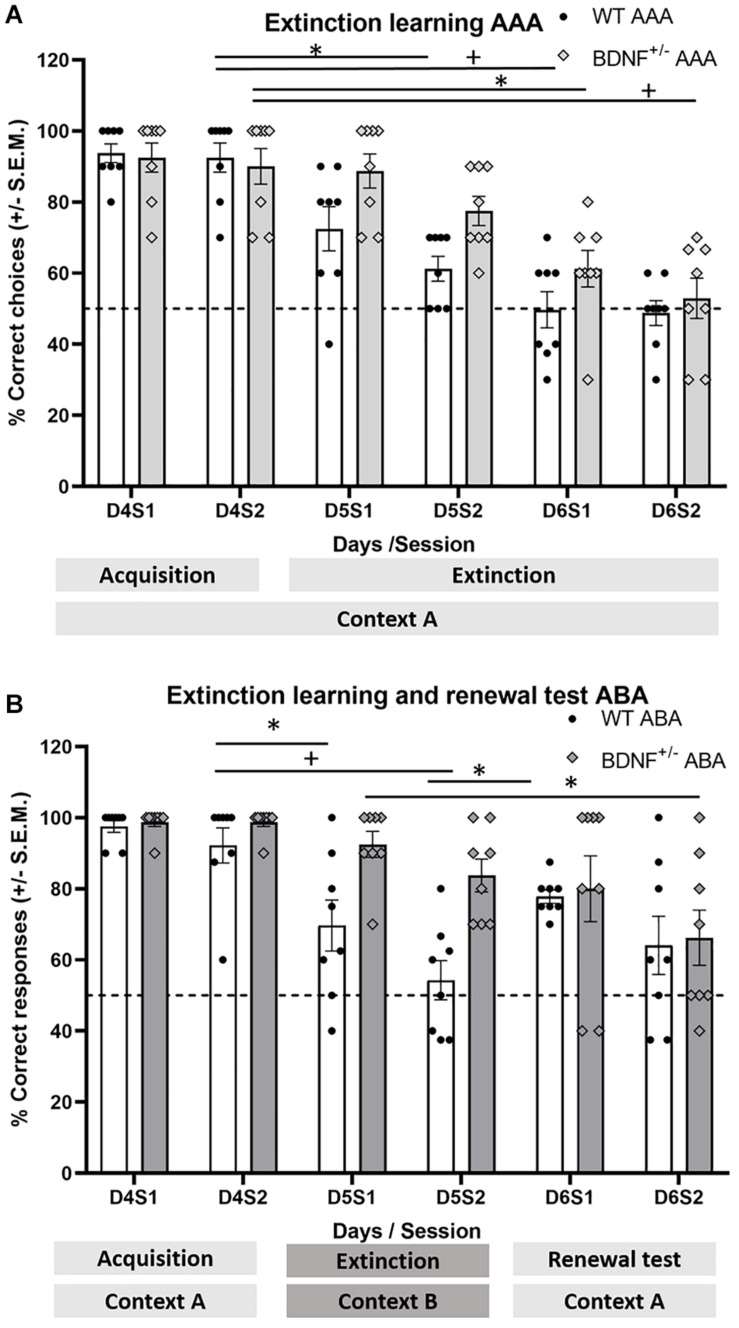
BDNF^+/−^ mice exhibit a delay in extinction learning in the AAA context and an impairment in extinction learning in the ABA context. The bar charts show the percentage of correct choices made by the animals during task acquisition on Day 4 involving sessions 1 and 2 in context A. In addition, arm choice performance is shown during the 2 days of extinction trials in the same context as used for acquisition (AAA paradigm) involving two training sessions per day (D5S1-D6S2). Furthermore, arm choice performance is shown when extinction trials occur in a novel context (compared to the acquisition conditions) during two sessions on Day 5 (D5S1, D5S2), and when animals are re-exposed to the original acquisition context during two sessions on Day 6 (D6S1, D6S2) (ABA paradigm). Each bar pair shows the animals’ correct choice performance. The dots (black: WT, gray outlined: BDNF^+/^) show the distribution of choice performance in a specific session, and the dashed line represents the chance performance level. **(A)** AAA paradigm: Although correct choices in BDNF^+/−^ mice (*n* = 8) and their WT littermates (*n* = 8) were equivalent during both acquisition learning sessions on Day 4 (D4S1, D4S2) when extinction learning was assessed on Day 5, a significant difference between extinction learning performance in WT and BDNF^+/−^ mice became apparent. WT mice showed rapid extinction learning (D5S2, D6S1), whereas effects in BDNF^+/−^ mice were significantly slower. By end of Day 6, BDNF^+/−^ mice exhibited extinction learning that was not significantly different from WT littermates, performing at chance levels (dashed line). **(B)** In the ABA paradigm, BDNF^+/−^ mice (*n* = 8) and their WT littermates (*n* = 8) also displayed equivalent levels of correct choices during both acquisition learning sessions in context A on Day 4 (D4S1, D4S2). However, when extinction learning was tested in context B on Day 5, a significant impairment in extinction learning became evident in BDNF^+/−^mice compared to WT littermates (whereas WT mice exhibit performance differences between the end of acquisition, D4S2 to D5S1 and D5S2, BDNF^+/−^ do not). Although WT mice exhibited significant renewal on Day 6 (correct choices during D6S1 compared to choices during D5S2), BDNF^+/−^ mice displayed choice levels during D6S1, that were not different from choices made on Day 5, indicating that WT exhibited renewal. By contrast, BDNF^+/−^ mice showed perseverance in entering the previously rewarded target arm. *(*p* ≤ 0.05) and ^+^(*p* ≤ 0.01).

To study the occurrence of extinction learning in the AAA paradigm, we compared the percentage of correct responses in a session by session manner and compared the last session of acquisition on day 4 with the extinction sessions on Day 5 and Day 6 in BDNF^+/−^ mice (*n* = 8) and WT littermates (*n* = 8). In the AAA paradigm, both groups reduced their percentage of correct responses and engaged extinction by the last session on Day 6 [*F*_(1,54)_ = 8.07, *p* < 0.01] (Figure 3A). However, whereas WT animals reduced their correct responses and engaged in extinction learning on day 5 of the extinction learning sessions [wt D4S2 vs. D5S2: *F*_(4,28)_ = 8.33, *p* < 0.01; *p* = 0.02], the BDNF^+/−^ mice needed an additional day to achieve a lower percentage of correct responses [BDNF^+/−^ D4S2 vs. D6S1: *F*_(4,28_) = 11.09, *p* < 0.01; *p* = 0.01] (Figure 3A).

### The Change of Context During Extinction Learning Improves Performance in Wild-Type but Not Transgenic Mice

When extinction learning in the absence (AAA) or presence (ABA) of the acquisition context was compared on Day 5, we detected significantly faster extinction learning in WT mice in the ABA paradigm (Two way ANOVA with session and group as factors [*F*_(3,56)_ = 14,76, *p* < 0.001], [Holm–Sidak test, used for all *post hoc* comparisons (*p* = 0.009)] (Figures 3A,B).

By contrast, we saw no difference in choice performance in the presence or absence of a context change when extinction learning was compared in BDNF^+/−^ mice on Day 5 (Figures 3A,B) (*p* = 0.324). Thus, extinction learning was not evident on day 5 in BDNF^+/−^ mice that were exposed to a context that was different from the acquisition context.

### Renewal Behavior Is Evident in Wild-Type Mice in the ABA Paradigm. BDNF^+/−^ Mice Show Gradual Extinction Learning

On Day 6 of the ABA paradigm, animals were returned to the acquisition context and correct choice behavior was assessed in the absence of a context change over a period of 20 trials (Figure 3B). Here, WT mice showed significant renewal behavior in the first 10 trials (session1) (D6S1 vs. D5S2, Holm–Sidak, *p* = 0.015). BDNF^+/−^ mice had not shown extinction learning behavior on Day 5 (Figure 3B). Thus, we assessed whether a return to the acquisition context on Day 6 had an influence on their behavior. Their initial response behavior was equivalent to their behavior on Day 5 (D6S1 vs. D5S2, Holm–Sidak, *p* = 0.051), but during the second session on Day 6, extinction learning emerged that was equivalent to behavior in WT animals (D6S2 vs. D5S2, Holm–Sidak, *p* = 0.024) and significantly different from choice behavior of the BDNF^+/−^ mice during the first session of Day 5 (D5S1 vs. D6S2, Holm-Sidak, *p* = 0.006).

Comparison of choice behavior on day 5 and 6 in the AAA and ABA paradigms for BDNF^+/−^ mice revealed differences between groups [*F*_(3,108)_ = 9.13, *p* < 0.001]. Specifically, differences were found between both WT groups when they performed in different paradigms (AAA vs. ABA) (Holm-Sidak test (*p* = 0.009)), also between BDNF^+/−^ mice performing in the two different paradigms (*p* = 0.008), revealing that the context change affects the performance for both genotypes.

The effect of the genotype was also measured in each paradigm, in the case of ABA, differences were found between experimental and control animals (WT in the ABA vs. BDNF^+/−^ in the ABA, *p* = 0.013) and the same occurred in the AAA paradigm, where differences between WT and BDNF^+/−^ mice were significant (*p* = 0.013).

All animals performed differently along the extinction and renewal test sessions, [*F*_(4,108)_ = 8.14, *p* < 0.001], specifically, between the beginning and the end of the unrewarded sessions (D5S1 vs. D6S2, *p* < 0.001) and between the beginning of Day 5 to beginning of Day 6 (*p* < 0.001) as well as among first and second session of Day 6 (*p* = 0.005).

Taken together, these data suggest that the change of context in the ABA paradigm did not accelerate extinction learning in BDNF^+/−^ mice. By contrast, it may have served to exacerbate this process, however, when the acquisition context was presented again on day 6, BDNF^+/−^ animals behave differently to those remaining in the B context.

### Trial-by-Trial Learning Scrutiny Reveals Impoverished Extinction Learning in the Acquisition Context and an Absence of Extinction Learning in a Changed Context in BDNF Transgenic Mice

**FIGURE 4:**
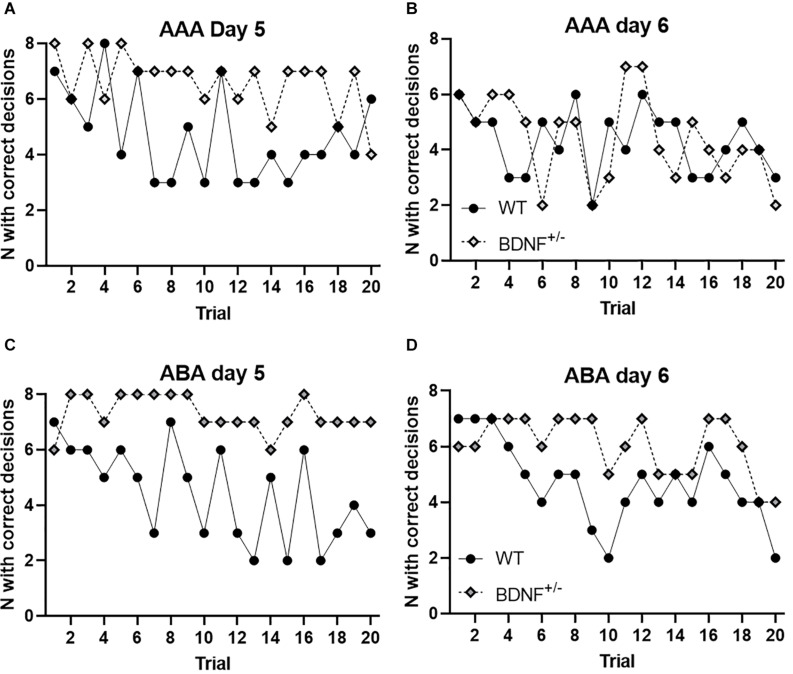
Comparison of trial-by-trial learning on days 5 and 6. To assess trial-by-trial learning, the number of correct arm choices made by each animal was plotted for each individual trial. **(A)** The number of animals entering the correct arm in context “A” on Day 5, consistently declined over the 20 trial period with regard to wild-type animals. By contrast, BDNF^+/−^ mice numbers remained consistently high. **(B)** The number of animals entering the correct arm in context “A” on Day 6 is equivalent in wild-type and BDNF^+/−^ mice. **(C)** When extinction learning is tested in context “B”, wild-type animals exhibit a rapid diminishment of choice behavior for the previously rewarded arm, whereas BDNF^+/−^ mice persist in entering the arm. **(D)** On day 6, animals were returned to the “A” context (in the absence of a reward). Here, WT animals show an increase of “correct” arm choices in the initial trials compatible with a renewal effect. This response then diminished corresponding to extinction learning as the animal understands that no reward will be made available. BDNF^+/−^ mice show arm choice behavior that is similar to Day 5, but toward the end of the trial block evidence of extinction learning begins to emerge.

To clarify to what extent putative differences in acquisition memory may have impacted on extinction learning performance, we examined trial-by-trial learning on days 5 and 6. In WT mice the first and second trial in the A or the B context elicited a very similar response (choice behavior of seven followed by six correct arm choices) (Figures 4A,C). This response was followed by a gradual decrease in entries into the formerly rewarded arm that was more effective in the condition where the context was changed (Figures 4A,C).

By contrast, BDNF^+/−^ mice showed a persistently high level of (previously) rewarded arm choices during the early trials on Day 5 (Figures 4A,C). In the AAA paradigm this choice behavior diminished slightly by the end of the trial block but remained significantly different from WT performance. In the ABA paradigm, no attrition in choice behavior was evident in throughout the entire duration of the trials on Day 5.

On Day 6, choice behavior in BDNF^+/−^ mice became increasingly similar to behavior in WT mice tested in the AAA paradigm (Figure 4B), but choice behavior was still significantly different in BDNF^+/−^ mice tested in the ABA paradigm (Figure 4D). Strikingly, choice behavior in BDNF^+/−^ mice on Day 6 in the ABA paradigm was not significantly different from choice behavior in transgenic mice on Day 5 in the AAA paradigm.

Taken together these data indicate that the acquisition representation may be more resilient and inflexible in BDNF^+/−^ mice compared to WT mice and that extinction learning is delayed in the absence of a context change in BDNF^+/−^ mice. Where extinction learning is conducted in a context that differs from the acquisition context, BDNF^+/−^ mice have greater difficulty in extinguishing the learned behavior. A return to the original acquisition context may ameliorate this extinction learning difficulty.

## Discussion

This study demonstrates that BDNF depletion is associated with an impairment of spatial appetitive extinction learning. Strikingly, extinction learning is delayed when it occurs in the original acquisition context and is potently impaired when it occurs in a context that differs from the acquisition context. Effects may derive from differences in information storage during acquisition learning along with a greater resistance in BDNF^+/−^ mice toward experience-dependent information updating.

The contribution of BDNF to associative learning and to hippocampus-dependent forms of memory is indeed experience-dependent (Aarse et al., 2016). In line with this, spatial reference memory in the water maze task is impaired when BDNF is unavailable (Minichiello et al., 1999). Most strikingly, BDNF is required for forms of hippocampal synaptic plasticity that are *directly triggered* by spatial learning. This suggests that BDNF plays a pivotal role in hippocampus-dependent experience encoding. In line with this, both BDNF^+/−^ mice and BDNF val66Met models exhibit deficiencies in contextual fear memory (Chen et al., 2006). Furthermore, in mice expressing promotor−IV of the BDNF gene, hippocampal BDNF expression is impaired and extinction learning of context−related fear association and perseverance are changed (Sakata et al., 2013; Sakata and Duke, 2014). We observed that although acquisition learning is ostensibly equivalent in BDNF^+/−^ mice and their WT littermates, extinction learning in the absence of a reward is impaired regardless of whether the animals undergo this process in the presence or absence of the original acquisition context. Trial-by-trial learning analysis revealed that BDNF^+/−^ mice were more persistent in looking for the reward in the (now unrewarded) target arm during extinction learning trials. By contrast, WT animals exhibited rapid behavioral adaptation that is fastest when extinction learning occurred in a novel context. Although spatial memory retrieval is hippocampus-dependent (Conejo et al., 2013; Kyle et al., 2015; Mendez-Couz et al., 2015) this finding suggests that BDNF-depleted mice may have used a different encoding strategy, or even different information encoding structures, for the acquisition of the correct arm-choice behavior that impacted on their retrieval of the acquisition memory in the early extinction learning trials, and may also have impacted on their ability to engage in extinction learning itself.

Spatial appetitive extinction learning recruits hippocampal gene encoding (Mendez-Couz et al., 2019). As yet, it is unclear to what extent this process involves hippocampal synaptic plasticity, but context-dependent forms of synaptic plasticity that are triggered by learning have been reported (Manahan-Vaughan and Braunewell, 1999; Kemp and Manahan-Vaughan, 2004). Long−term hippocampus−dependent memory is associated with the expression of persistent forms of LTP (Kemp and Manahan-Vaughan, 2004; Novkovic et al., 2015) and LTD (Kemp and Manahan-Vaughan, 2004, 2007, 2012; Kemp et al., 2013). Both learning−facilitated LTD and spatial reference memory are impaired in BDNF^+/−^ mice (Novkovic et al., 2015; Aarse et al., 2016), suggesting that it is critically required for the physiological encoding of hippocampus−dependent memory.

The hippocampus is involved in appetitive spatial extinction learning in an ABA T-maze paradigm (Mendez-Couz et al., 2019). Peters and colleagues (Peters et al., 2010) proposed that BDNF may be transported from the hippocampus to the infralimbic cortex to facilitate the extinction of fear memory, given that the hippocampus projects to the infralimbic cortex (Hoover and Vertes, 2007). By contrast, others (Rosas-Vidal et al., 2018) reported a BDNF expression increase in the ventral hippocampus, but not in the infralimbic cortex, following fear-conditioned extinction training. This may relate to the fact that inactivation of the prelimbic and infralimbic areas of the mPFC impair memory for multiple task switches, but not for the flexible selection of familiar tasks (Rich and Shapiro, 2007). Interestingly, flexible spatial learning depends on both the dorsal and ventral hippocampus and their functional interactions with the mPFC (Avigan et al., 2020). Thus, the apparent deficits in information updating apparent in the BDNF^+/−^ mice in the present study may relate to a deficiency in BDNF-support of learning flexibility and task switching that is mediated by impaired information encoding in both the hippocampus and prefrontal cortex (Olsen et al., 2013). This property may also explain why the BDNF^+/−^ mice continued to return to the previously rewarded arm during the extinction learning trials.

Context-dependent learning results in rapid induction of BDNF in the hippocampus (Hall et al., 2000). The depletion of BDNF in the transgenic animals that were used in the present study will have resulted in less BDNF induction during the change of T-maze context. This is likely to have undermined context-dependent information updating both when extinction learning occurred in the acquisition context, and when it occurred in a new context. Consistent with this interpretation is the finding that local depletion of BDNF within the dorsal hippocampus prevents spatial learning and fear extinction learning without affecting information acquisition (Heldt et al., 2007). This does not of course exclude that depleted BDNF in other brain regions contributed to these deficits (Gorski et al., 2003).

Re-activation of a memory may also require both the hippocampus and the prefrontal cortex. Eddy et al. (2016) proposed that there is a region of medial prefrontal cortex encompassing both dorsomedial prefrontal cortex and ventromedial prefrontal cortex that is important for ABA renewal of extinguished operant conditioning of a food reward. This study suggests that the dorsomedial prefrontal cortex plays a role in ABA renewal of extinguished operant response (lever pressing) and that inactivation of the ventromedial PFC impairs both extinction learning and ABA renewal expression (Vasquez et al., 2019). Due to the fact that our BDNF^+/−^ mice did not engage in extinction learning in the “B” context on Day 5, their relatively high level of correct arm choices during re-exposure to the “A” context on Day 6 cannot be interpreted as a renewal/re-activation response. Here, we assume that the correct arm choices reflect perseverance in searching for a reward in the previously rewarded arm, consistent with a BDNF-mediated impairment in updating the spatial representation and association. By contrast, WT animals exhibited significant renewal behavior on Day 6.

The impairment of extinction learning both in the presence and absence of a context change that we observed in BDNF^+/−^ mice may derive from the requirement of BDNF for memory updating. The persistent search behavior of the previously rewarded arm seen in BDNF^+/−^ mice during the early extinction learning trials suggests that BDNF depletion subverted the ability of the animals to update their representation of the T-maze environment and the association of a specific maze arm with a reward. In line with this, a role for BDNF has been proposed in behavioral perseverance (Sakata et al., 2013). The fact that the extinction learning deficits were apparent both in the presence and absence of a context change, although information acquisition was not affected, suggests that extinction learning conducted in either context involves information updating, rather than *de novo* encoding, and indicates that both processes require BDNF-mediated cell signaling. Here, too it has been proposed that BDNF is required for spatial reversal learning (Sakata et al., 2013).

It is quite intriguing that the presence of a context change may have slowed the ability of the animals to engage in extinction learning. Three possible interpretations of the absence of extinction learning in the “B” context spring to mind: (1) The BDNF^+/−^ animals did not register the context change and/or its saliency and thus behaved exactly like the BDNF^+/−^ mice that were tested for extinction learning in the “A” context. This impairment would have presumably been mediated by impairment of hippocampal information processing and context-specific updating (Matus-Amat et al., 2004; Takahashi, 2013; Radiske et al., 2017). (2) The presence of the novel context served as a distractor for the learning-impaired BDNF^+/−^ mice, thus counteracting the typical beneficial effects of a context-change on extinction learning (André et al., 2015b). This effect is likely to have been mediated by disrupted information processing in the prefrontal cortex (Barbas and Zikopoulos, 2007; Cosman et al., 2018; Yang and Mailman, 2018). (3) Working memory was disrupted in the BDNF^+/−^ mice, such that the animals did not remember that that the arms they entered did not contain a reward. This is an attribute ascribed to the prefrontal cortex and which is regulated by BDNF (Galloway et al., 2008). Cooperation between both structures is required for adaptive spatial memory updating and working memory, however, indicating that the BDNF depletion is likely to have affected extinction learning through impoverishing information processing in both structures (Sakata et al., 2013; Spellman et al., 2015; Hasz and Redish, 2020).

Previously, we reported no change in proBDNF levels in BDNF^+/−^ mice (Novkovic et al., 2015). Thus, the likely candidate in this process is the TrkB-mediated receptor pathway that is activated preferentially by mature BDNF (Lee et al., 2001; Ibánez, 2002; Teng et al., 2005) and leads to activation of MAP kinase and phosphatidylinositol-3-kinase (PI3K) (Kaplan and Miller, 2000; Patapoutian and Reichardt, 2001; Sweatt, 2001; Huang and Reichardt, 2003). This signaling pathway has been proposed to enable the integration of newborn cells into hippocampal networks (Bergami et al., 2008) that then improve the robustness of a stored representation. The BDNF/TRkB pathway is also intrinsically involved in hippocampal synaptic plasticity and memory (Snyder et al., 2001; Bruel-Jungerman et al., 2007; Sisti et al., 2007).

## Conclusion

We report here, that extinction learning in a context that differs from the acquisition context is more sensitive to the reduced availability of BDNF than extinction learning in the acquisition context. In the latter, AAA paradigm, extinction learning was delayed, whereas, in the former ABA paradigm, extinction learning was potently impaired in comparison to WT littermates. Both impairments occurred despite the fact that acquisition learning was successful in BDNF knockdown. However, trial-by-trial learning assessments suggest that information acquisition may have nonetheless resulted in either different representations or more inflexible representations in BDNF^+/−^ mice. This means that novel spatial learning *per se* can occur in BDNF^+/−^ mice, but information updating related to extinction learning is disrupted. In both the AAA and ABA paradigms, animals persisted in looking for the reward in the target arm, even though it was no longer present (and had been removed prior to the extinction learning trials), indicating that inappropriate perseverance occurred. Furthermore, the presence of context change failed to support extinction learning in BDNF^+/−^ mice, and may even have served to exacerbate it. Taken together these findings suggest that BDNF is required for learning flexibility and information updating related to spatial and extinction learning.

## Data Availability Statement

The raw data supporting the conclusions of this article are available from the corresponding author, upon reasonable request.

## Ethics Statement

The animal study was reviewed and approved by Landesamt für Arbeitschutz, Naturschutz Umweltschutz und Verbraucherschutz (LANUV) Bezirksamt, Arnsberg, Germany.

## Author Contributions

DM-V designed the study. MM-C conducted the behavioral experiments. BK conducted the PCR and Western Blot. MM-C and DM-V wrote the manuscript. All authors analyzed the data.

## Conflict of Interest

The authors declare that the research was conducted in the absence of any commercial or financial relationships that could be construed as a potential conflict of interest.
